# Is why we drink alcohol important when considering the potential public health benefit of alcohol-free and low-alcohol drinks? A cross-sectional study investigating associations between alcohol drinking motives and alcohol-free and low-alcohol drink consumption among adults in Great Britain

**DOI:** 10.1136/bmjph-2025-002828

**Published:** 2025-09-08

**Authors:** Lucy C Burke, Colin Angus, Jamie Brown, Inge Kersbergen

**Affiliations:** 1 Sheffield Centre for Health and Related Research (SCHARR), University of Sheffield, Sheffield, UK; 2 Public Health and Epidemiology, University College London, London, UK; 3 Department of Psychology, University of Bath, Bath, UK

**Keywords:** Public Health, Cross-Sectional Studies, Sociodemographic Factors, statistics and numerical data

## Abstract

**Introduction:**

The UK has promoted increased availability of alcohol-free and low-alcohol drinks (no/lo, ≤1.2% alcohol by volume) as a public health strategy. To be effective, no/lo beverages must replace, and not supplement, standard alcoholic drinks. Emerging evidence suggests that the reasons people drink alcohol may be an important determinant of the potential public health impact of these drinks. This study aimed to determine whether alcohol drinking motives were associated with no/lo consumption after accounting for sociodemographic characteristics and alcohol consumption.

**Methods:**

A cross-sectional sample of adults residing in Great Britain (aged 16–93) who had drunk alcohol in the past year were recruited via the Alcohol Toolkit Study (N=2555; 49.0% female). The dependent variable was frequency of no/lo consumption (less than vs at least monthly). Five questions captured respondents’ alcohol drinking motives (enhancement, social, conformity, coping-anxiety, and coping-depression), derived from the Drinking Motives Questionnaire-Revised. Sociodemographic characteristics, including age, gender, social grade, education, Index of Multiple Deprivation (a UK-wide measure of relative deprivation for small geographic areas), and hazardous alcohol use (Alcohol Use Disorder Identification Test), were also assessed. Descriptive analysis presents the proportion of respondents drinking no/lo at least monthly among low endorsement (ie, drinking for a motive less than half the time) versus high endorsement (ie, drinking for a motive at least half the time) of each drinking motive. Quasibinomial regression modelling explored relationships between alcohol drinking motives and no/lo consumption, accounting for sociodemographic characteristics and hazardous drinking.

**Results:**

Drinking alcohol to conform was associated with an increased likelihood of at least monthly no/lo consumption after accounting for sociodemographic characteristics and hazardous drinking (OR 1.10, 95% CI 1.00 to 1.21, p=0.041).

**Conclusions:**

No/lo drinks may facilitate reduced alcohol consumption by offering an alternative for individuals wishing to participate in alcogenic environments. However, those who drink alcohol to conform are not typically higher-risk drinkers, which may limit the public health benefit of no/lo drinks. Further research is needed to explicitly explore substitution effects.

**WHAT IS ALREADY KNOWN ON THIS TOPIC**

- Since 2019, alcohol-free and low-alcohol (no/lo) drinks have been endorsed by successive UK governments as a public health strategy.
- Qualitative studies indicate that the reasons people drink alcohol may be important when investigating whether no/lo drinks are an acceptable substitute to standard alcohol.

**WHAT THIS STUDY ADDS**

- This is the first study to quantitatively explore whether the reasons people drink alcohol are associated with no/lo consumption.
- Respondents who reported drinking alcohol to conform were more likely to report drinking no/lo at least monthly after controlling for sociodemographic characteristics and hazardous drinking.

**HOW THIS STUDY MIGHT AFFECT RESEARCH, PRACTICE OR POLICY**

- Further research is needed to explicitly explore substitution effects. To maximise the public health benefit of the ‘no/lo policy’, additional strategies may be required to encourage some at-risk drinking groups to substitute standard alcohol with no/lo alternatives.

## Introduction

 Reducing alcohol harm is a critical public health priority in the UK. While growing numbers of drinkers seek to moderate their consumption, a large minority is drinking alcohol at increasingly harmful levels.^1 2^ This could lead to a 20% increase in alcohol-related mortality over the next 20 years, costing the National Health Service (NHS) up to £5.2 billion.^3^

Increasing the availability of alcohol-free and low-alcohol (no/lo) drinks has been promoted as a public health strategy by the UK Department of Health and Social Care.^4 5^ Since 2015, there has been a proliferation of these products emerging onto the UK market and elsewhere, with further growth predicted.^6^,^8^ If consumers can be encouraged to substitute standard alcohol with no/lo alternatives, this could lead to a public health benefit.^4 9^

In the UK, no/lo drinks are defined as alcoholic or alcoholic type (eg, beer, wine, spirits) drinks that contain ≤1.2% alcohol by volume (ABV)^10^, a threshold which aligns with current UK alcohol duty rates.^11^ They do not include soft drinks or lower-strength alcoholic drinks that have an ABV above 1.2% ABV. While some no/lo beverages include a small amount of alcohol, they are unlikely to lead to intoxication.^12^ In Great Britain, while currently not illegal, there is a voluntary agreement among alcohol licence holders that no/lo drinks are not sold to individuals aged under 18, in line with the legal age for purchasing alcoholic drinks.

Given that the objective of the ‘no/lo policy’ is for drinkers to substitute alcohol with no/lo, it is pertinent to consider the reasons why people drink alcohol and how well no/lo drinks may satisfy these motives.^13 14^ People drink alcohol for many reasons: alcohol can signify celebration, serve as a social lubricant and make people feel happy.^15 16^ Some people use alcohol to self-medicate, believing it will help them cope with anxiety or depression.^17^,^19^ In many countries, including the UK, alcohol is central to social culture.^20^ Both academics and policy-makers support a consideration of alcohol drinking motives when developing alcohol reduction interventions.^1421^,^23^

Cox and Klinger’s^24^ motivational model of alcohol use places alcohol drinking motives along two dimensions.^24^ First, motives are identified as having an internal (the self) or external (social environment) source, and second, motives are driven by positive (eg, drinking alcohol for the buzz, making social occasions more enjoyable), or negative (eg, coping with low mood or anxiety, conforming to expectations) reinforcement. Several measures of drinking motives exist.^18 25 26^ Cooper’s Drinking Motives Questionnaire (DMQ) captures drinking alcohol for enhancement, conformity, social and coping reasons and is a widely used and well-validated tool.^25^ Its psychometric properties have been tested in multiple countries, and importantly, on adult populations.^27^,^30^

The emerging literature regarding no/lo consumption supports the idea that considering alcohol drinking motives is important. Studies have found that no/lo consumers acknowledge social participation and adhering to social norms as key benefits of no/lo.^31^,^35^ An Australian qualitative study of adults who had reduced their alcohol consumption reported alcohol-free drinks allowed participants to masquerade as ‘drinkers’, a key strategy in their successful reduction attempts, allowing them to remain aligned with cultural expectations.^32^ Studies conducted in the UK, including consumers of no/lo drinks, and pregnant women, found no/lo drinks facilitated social occasions, enabling participation where alcohol consumption was typical, and allowing those not drinking alcohol to avoid scrutiny from peers.^31 33 36^ Conversely, studies including those who did not consume no/lo drinks found that these respondents often did not see the point of no/lo drinks if the goal was to feel inebriated.^33^,^3537^

These studies suggest that those who drink for external reasons, particularly what is defined as ‘conformity’ in Cox and Klinger’s motivational model of alcohol use,^24^ may be more likely to consume no/lo drinks than those who drink for internal reasons. This is concerning because ‘internal drinkers’ are most at risk of alcohol harm.^38^ Furthermore, those drinking to cope are likely to be less socially advantaged,^21^ a group less likely to consume no/lo drinks.^39^ To date, there are no studies which have explicitly explored whether the reasons people drink alcohol are associated with no/lo consumption using quantitative methods. This is important from a public health perspective where we are specifically interested in considering how well no/lo drinks may encourage a reduction in alcohol consumption, rather than looking to understand no/lo behaviour more generally.

### The present study

The current study uses data from a nationally representative survey of adults residing in Great Britain to explore: (1) whether there are direct associations between alcohol drinking motives and no/lo consumption and (2) whether alcohol drinking motives help to explain no/lo consumption after accounting for sociodemographic characteristics. The study addressed the following hypotheses:

People who endorse drinking alcohol for internal reasons (enhancement and to cope with anxiety or depression) will have significantly lower odds of consuming no/lo drinks at least monthly than those who do not drink alcohol for these reasons after accounting for sociodemographic characteristics and hazardous drinking. We did not expect an association between external motives and no/lo consumption. We also expected to find a higher odds of regular no/lo consumption among those who were socially advantaged, assessed using measures of social grade and highest level of education received, and higher risk drinkers, as found with previous work.^39^ We explored whether neighbourhood level deprivation was associated with no/lo consumption, using the Index of Multiple Deprivation (IMD).^40^

## Method

### Design

A cross-sectional study of adults aged 16 and over, recruited via the February 2023 and April 2023 waves of the Alcohol Toolkit Study.^41^ This is a monthly telephone survey of adults residing in Great Britain, capturing respondents’ alcohol drinking behaviour. The sampling process aims to recruit a study population that is nationally representative in terms of gender, working status, prevalence of children in the household, age, social grade and region.^41^ A rim (marginal) weighting technique is used to ensure the target profiles are met.^42^ Alongside routinely administered questions capturing respondent demographics and alcohol use, respondents also reported how often they consumed no/lo drinks. In these two waves only, five additional questions capturing respondents’ alcohol drinking motives were included.

### Sample

Across the two waves, there were 2920 respondents who had drunk alcohol at least once in the previous 12 months as recorded by the Alcohol Use Disorder Identification Test-C (AUDIT-C).^43^ After removing those whose responses made them ineligible for inclusion, there remained a sample of 2555. This sample of 2555 included 440 cases with missing data, typically single items, which had then been imputed to provide a complete dataset (see online supplemental figure 1 for a participant flow chart and Analysis for further detail). The weighted sample was 2597. This was powered to detect ORs greater than 1.15 at 80% power and 5% alpha in a logistic regression.^33^

### Measures

#### No/lo drinking behaviour

We measured frequency of no/lo consumption as a single item.^39^ Participants were asked, “How often do you have an alcohol-free or low-alcohol drink (beer, wine, cider, spirits or other type of alcoholic drink under 1.2% ABV)?”. Participants responded on an 8-point scale, ranging from never to nearly every day. Due to low numbers responding at higher frequencies, responses were recoded as a binary variable–less than monthly/at least monthly, to capture whether respondents were a regular consumer of no/lo drinks or not.

#### Alcohol drinking motives

Alcohol drinking motives were captured using five items from Cooper DMQ Revised (DMQ-R).^25^ This measure captures emergent themes from the qualitative literature around no/lo consumption and has been validated in several countries, including England, and on adult populations.^27 30^ Due to financial constraints, single items were chosen to capture each alcohol drinking motive. Single items have been used to capture alcohol drinking motives, including the motives captured in the DMQ-R, elsewhere.^44 45^ We also chose to distinguish between coping-anxiety and coping-depression by selecting two items from the coping subscale which represent these different aspects of coping. This distinction was made due to evidence that these motives are differentially associated with drinking patterns and socioeconomic status.^18 46^ While the modified DMQ-R distinguishes between these two motives, its authors note it has unsatisfactory psychometric properties for its social scale and has not yet been validated on adults.^18^ Therefore, we chose to use items from the DMQ-R.^25^

Item selection was informed by each item’s psychometric properties and patient and public involvement (PPI, see PPI statement). The selected items were:

1. Because it gives you a pleasant feeling (Enhancement).
2. Because it makes social gatherings more fun (Social).
3. To fit in with a group that you like (Conformity).
4. Because you feel more self-confident and sure of yourself (Coping-anxiety).
5. To forget about your problems (Coping-depression).

Responses were recorded on a 5-point scale (1=never/almost never, 2=some of the time, 3=half of the time, 4=most of the time, 5=almost always/always). Alcohol drinking motives were treated as continuous variables in the main analyses, but to aid interpretation in the descriptive analysis they were presented as binary variables (responses of never, almost never and some of the time=low endorser, responses of half the time, most of the time and almost always/always=high endorser).

#### Harmful alcohol consumption

The AUDIT-C measured hazardous alcohol consumption.^43^ It discriminates between those at higher or lower risk of alcohol-related harm. A three-item scale captures frequency of alcohol consumption, numbers of units of alcohol consumed during a typical drinking occasion and frequency of heavy episodic drinking (six or more units of alcohol in a single drinking occasion). Responses were recoded to correspond with validated AUDIT-C scoring to produce a total score between 0 and 12, treated as a continuous variable. Non-drinkers were excluded; therefore, scores in the study sample ranged from 1 to 12.

#### Sociodemographic variables

The routinely collected variables in the ATS that were used in the analysis included:

- Age (16–24, 25–34, 35–44, 45–54, 55–64, 65+).
- Gender (male, female).
- Highest level of education attained (secondary school education or equivalent; preuniversity qualification, for example, A-levels, International Baccalaureate Diploma or equivalent; bachelor’s degree or equivalent undergraduate degree; postgraduate qualification or equivalent).
- Social grade (AB=higher/intermediate managerial, administrative or professional, C1=supervisory, clerical and junior managerial, administrative or professional, C2=skilled manual workers, DE=semiskilled and unskilled manual workers, pensioners, casual and lowest grade workers, unemployed and in receipt of state benefits only^47^).
- IMD based on a respondent’s postcode. IMD captures local level data on income, health, education, crime, environment, barriers to housing and living environment. Five response levels range from: 1=most deprived quintile to 5=least deprived quintile.^40^

Age, social grade and education were treated as factors, whereas IMD was treated as a continuous variable. Ethnicity is reported descriptively (white, black, Asian, mixed heritage, other, table 1) but was not included in the regression model due to small numbers of black, Asian and other ethnically diverse groups in the sample population.

**Table 1:** Sample characteristics (weighted, n=2597)

Characteristic	Statistic
No/lo consumption	n (%)
At least monthly	550 (21.2)
Less than once a month	2047 (78.8)
Drinking motives (ordinal)	M, (SD, range, 95% CI)
Enhancement	2.71 (1.44,1 to 5, 2.65 to 2.77)
Social	2.64 (1.38, 1 to 5, 2.58 to 2.70)
Conformity	1.61 (1.07, 1 to 5, 1.56 to 1.65)
Coping-anxiety	1.60 (1.07, 1 to 5, 1.55 to 1.65)
Coping-depression	1.30 (0.80, 1 to 5, 1.27 to 1.34)
Drinking motives (at least half the time/high endorsers)	n (%)
Enhancement	1158 (44.6)
Social	1105 (42.5)
Conformity	352 (13.6)
Coping-anxiety	362 (13.9)
Coping-depression	164 (6.3)
Hazardous alcohol consumption	Mean (SD, 95% CI)
AUDIT-C	4.36 (2.54, 4.25 to 4.67)
AUDIT-C score risk classifications	n (%)
Low risk (scores 0–4)	1551 (59.7)
Increasing risk (scores 5–7)	687 (26.5)
Higher risk (scores 8–10)	321 (12.4)
Possible dependence (score 11–12)	38 (1.5)
Age	n (%)
16–24	308 (11.8)
25–34	414 (15.9)
35–44	440 (16.9)
45–54	455 (17.5)
55–64	545 (21.0)
65+	545 (21.0)
Gender*	n (%)
Male	1325 (51.0)
Female	1272 (49.0%)
Social grade*	n (%)
AB (higher or intermediate managerial)	782 (30.1)
C1 (supervisory/clerical, junior managerial administrative/professional)	769 (29.6)
C2 (skilled manual)	554 (21.3)
DE (semikilled/unskilled manual, casual or lowest grade, pensioners, others who depend on the welfare state for their income).	492 (18.9)
Highest level of education attained	n (%)
Secondary school/equivalent	673 (25.9)
College (A Levels)/equivalent	668 (25.7)
Undergraduate degree/equivalent	822 (31.7)
Postgraduate degree/equivalent	434 (16.7)
IMD quintile*†	n (%)
1 (most deprived)	431 (16.6)
2	502 (19.3)
3	552 (21.2)
4	555 (21.4)
5 (least deprived)	558 (21.5)
Ethnicity	n (%)
White British/other	2327 (89.6)
Black British/other	96 (3.7)
Asian British/other	65 (2.5)
Mixed heritage	62 (2.4)
Other ethnicities including not specified	47 (1.8)

* Uses imputed estimates where values were missing.

† IMD captures local-level data on income, health, education, crime, environment, barriers to housing and living environment to produce a measure of relative deprivation. Five response levels range from: 1=most deprived quintile to 5=least deprived quintile.^40^

AUDIT-C, Alcohol Use Disorder Identification Test-C; IMD, Index of Multiple Deprivation; No/lo, alcohol-free and low-alcohol.

### Patient and public involvement

The Alcohol Toolkit Study is a well-established survey, therefore, PPI work focused on the selection of outcome measures specifically added for this study. Seven members of the University of Stirling’s Alcohol and Food Discussion Group (https://spectrum.ed.ac.uk/about/public-involvement), an established PPI group that supports research in this area, assisted in selecting items from the DMQ-R to be included in the survey. In response to participant preferences, the PPI meeting was held online. Participants brought their own lived experience with regards to alcohol consumption to the discussion. All participants drank alcohol at least occasionally, and the group comprised both those who did and did not consume no/lo drinks.

Following a general introduction and warm-up session about no/lo drinks, participants were asked to contribute to: (1) a general discussion of the reasons why they drank alcohol and (2) a discussion about how well they felt the shortlisted alcohol drinking motives captured each of the overarching alcohol drinking motives. The group supported the selection of the shortlisted items for enhancement, coping-anxiety, coping-depression, and social subscales. For the conformity subscale, the group recommended an alternative item. The recommended item had good factor loadings and face validity; therefore, the shortlisted item for conformity was replaced to reflect the views of the PPI group.

In addition to academic dissemination of the findings of this study, dissemination with the wider public is ongoing. Preliminary findings have been shared at two public events: (1) a Pint of Science event in 2024 (https://pintofscience.co.uk/, https://pintofscience.co.uk/event/mocktails-and-chemtrails) and (2) a webinar run by the University of Sheffield that was advertised and accessible to all (https://www.sheffield.ac.uk/alumni/bright-minds) Further dissemination with the public, including those involved in the PPI work and relevant stakeholders, is ongoing.^48^

### Preregistration

The study’s analytical plan was preregistered on the Open Science Framework (osf.io/6rn3w). The analysis plan documents the planned analysis presented here and an additional path analysis which will be published separately. Changes to the analytical plan included:

- Analyses exploring location and rurality were not pursued. This decision was based on a recently published analysis,^40^ which used a larger dataset from the same source and yielded inconclusive findings. We determined that a similar analysis with our smaller sample would be unlikely to provide meaningful insights. Analyses exploring direct relationships between alcohol drinking motives, sociodemographic characteristics, hazardous drinking and no/lo consumption were combined into a single regression model.
- Analyses were population weighted.
- Regression models using rank ordering are not presented. We had been interested in exploring whether both relative and absolute endorsement of the alcohol drinking motives were important. However, very few respondents rated drinking alcohol for depression (n=31, 1.2%), anxiety (n=50, 2.0%), and conformity (n=94, 3.7%) as their primary motive, meaning this analysis was not possible.
- A sensitivity analysis using alcohol drinking motives recoded as binary variables (low vs high endorsers) was included.

### Analysis

Data preparation and analyses were undertaken in R V.4.3.1.^49^ The following groups of respondents were removed:

1. Respondents who answered inconsistently regarding their no/lo consumption (ie, responding that they engaged in situation-specific no/lo consumption: hybrid, on-trade or off-trade more often than they reported consuming no/lo drinks overall, n=163). This follows good practice advice for data cleaning^50^ and aligns with practice used in other studies reporting on this data.^39^
2. Respondents who reported that they did not know whether they drank alcohol for any of the drinking motives (n=189). While a debate exists as to whether ‘don’t know’ responses should be treated as missing, or identified as a substantive response,^51^ for our research we chose to exclude these participants. Individuals providing a ‘don’t know’ response for the drinking motives did not differ from the rest of the sample on key demographic variables (age, sex, social grade, education level, alcohol consumption, no/lo consumption).
3. Respondents describing their gender in another way (n=13). This final group was removed due to their small number, meaning it was not possible to meaningfully include them in the analysis.

Complete data were available for 2118 of 2555 respondents (82.9%). A flow chart illustrating participant eligibility and missing data is presented in online supplemental figure 1. The following variables had missing data: gender, n=6; social grade, n=106, IMD, n=358. Little’s missing completely at random (MCAR) test was significant, indicating it was not appropriate to treat data as MCAR.^52^ By investigating patterns of missing data, there was no evidence of systematic missingness; therefore, we felt it was appropriate to assume the data was missing at random and proceeded with multiple imputation, using the mice package in R.^53 54^ 18 datasets were imputed. Trace plots of the means and SD of the imputed values for the variables with missing data (IMD, social grade, sex) indicated that the imputation chains converged well. The primary analyses present pooled results from the imputed datasets which were then population weighted. The impact of survey weighting was evaluated (online supplemental table 1). It appeared to effectively adjust the sample to better represent the target population without unduly distorting key variable means.

#### Descriptive analysis and regression modelling

Descriptive analyses illustrate the proportions of respondents consuming no/lo drinks at least monthly for low and high endorsers of each alcohol drinking motive. Quasibinomial logistic regression models, including drinking motives as continuous variables, tested for associations between regular no/lo consumption (dependent variable) and alcohol drinking motives. This method is a robust approach for binary outcomes when overdispersion is present,^55 56^ which was a concern given the low base rate of at least monthly no/lo consumption (21%) in our sample. While negative binomial or zero-inflated regression models are valuable for addressing overdispersion, they are primarily designed for count data rather than the binary (yes/no) outcome capturing no/lo consumption in this study. The quasibinomial approach, which models a dispersion parameter, was thus the most appropriate method to account for overdispersion while maintaining the binary nature of our dependent variable.

The unadjusted regression model included drinking motives and no/lo consumption. The adjusted model controlled for sociodemographic characteristics (gender, age, education, social grade, and IMD) and hazardous drinking (AUDIT-C). Ordinal variables (age, education and social grade) were presented as factors. All analyses were population weighted and tests for the key assumptions of this analysis were undertaken.^57^ The data breached the linearity of log-odds assumption for AUDIT-C; therefore, an exploration of higher polynomial terms for AUDIT-C was undertaken. This indicated that AUDIT-C had a quadratic relationship with the dependent variable; consequently, a linear and quadratic term for AUDIT-C was included in the model. There was no evidence of multicollinearity among independent variables using variance inflation factors (online supplemental Table 2). The discriminative power of the primary model was assessed using receiver operating characteristic area under the curve (AUC).

#### Sensitivity analyses

Two sensitivity analyses were undertaken: (1) using complete cases and (2) including alcohol drinking motives coded as binary variables (low vs high endorsers).

## Results

### Participant characteristics

A summary of the weighted study sample is provided in table 1. 21% of respondents were consuming no/lo drinks at least monthly (n=550). Respondents were most likely to report drinking alcohol for enhancement and social reasons and least likely to report drinking alcohol to cope with depression. 12% of respondents (n=306) reported never drinking alcohol for any of the motives presented.

#### Exploring associations between alcohol drinking motives and no/lo consumption

**Figure 1:**
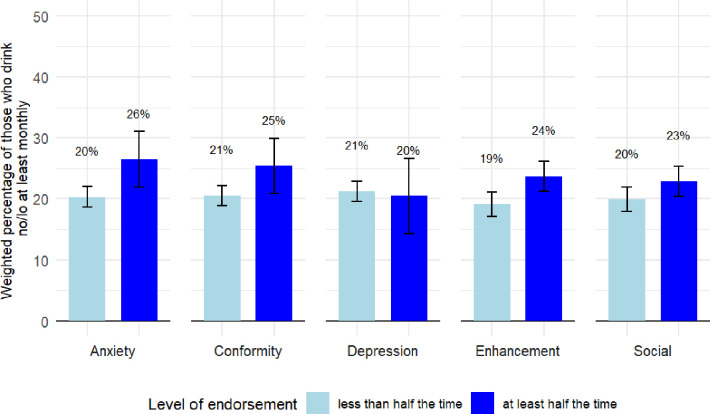
The percentage of low and high endorsers of each alcohol drinking motive who reported regular no/lo consumption, with 95% CIs, weighted (n=2597).

Figure 1 compares the proportion of respondents consuming no/lo drinks at least monthly for low and high endorsers of each alcohol drinking motive. Across all alcohol drinking motives, approximately 20% of low endorsers reported consuming no/lo drinks at least monthly (range: 19.1%–21.2%). Among high endorsers of each motive, no/lo consumption ranged from 20.5% for those drinking to cope with depression, to 26.5% for those drinking to cope with anxiety (online supplemental table 3).

The unadjusted quasibinomial logistic regression revealed that among the alcohol drinking motives assessed, only the enhancement motive was significantly associated with the likelihood of consuming no/lo drinks at least monthly (OR=1.09, 95% CI (1.01 to 1.18), p=0.030). For every unit increase in the enhancement motive score, the odds of consuming no/lo drinks increased by approximately 9%, while holding other drinking motives constant. The remaining motives were not significantly related to no/low alcohol consumption (table 2).

**Table 2:** Associations between regular no/lo consumption and alcohol drinking motives (weighted, n=2597)

Indicator	OR	95% CI	P value
(Intercept)	0.18	0.13 to 0.24	0.000
**Enhancement**	**1.09**	**1.01 to 1.18**	0.030*
Social	0.97	0.89 to 1.06	0.503
Conformity	1.10	0.99 to 1.21	0.072
Anxiety	1.08	0.96 to 1.20	0.190
Depression	0.99	0.86 to 1.13	0.874

Significant relationships (<0.05) are highlighted in bold.

* p<0.05.

no/lo, alcohol-free and low-alcohol.

In the adjusted model, enhancement was no longer significantly associated with no/lo consumption. In this model, drinking alcohol to conform was the only motive significantly associated with at least monthly no/lo alcohol consumption (OR=1.10, 95% CI (1.00 to 1.21), p=0.041, table 3). For every one-unit increase in the conformity motive score, the odds of consuming no/lo drinks at least monthly increased by approximately 10%, assuming all other variables in the model were held constant. The remaining drinking motives did not show a significant association with no/lo consumption in this model.

**Table 3:** Associations between regular no/lo consumption and alcohol drinking motives, after accounting for sociodemographic characteristics and alcohol consumption (weighted, n=2597)

Indicator	OR	95% CI	P value
(Intercept)	0.08	(0.04 to 0.14)	0.000
Enhancement	1.03	(0.95 to 1.11)	0.532
Social	0.94	(0.86 to 1.02)	0.131
**Conformity**	**1.10**	(**1 to 1.21**)	0.041*
Anxiety	1.09	(0.99 to 1.21)	0.092
Depression	1.02	(0.9 to 1.15)	0.799
**AUDIT-C (linear**)	**1.41**	(**1.21 to 1.64**)	0.000***
**AUDIT-C (quadratic**)	**0.98**	(**0.96 to 0.99**)	0.000***
Women (compared with men)	0.86	(0.71 to 1.05)	0.149
Age 25–34†	0.94	(0.64 to 1.38)	0.756
Age 35–44†	1.00	(0.68 to 1.47)	0.985
Age 45–54†	0.88	(0.6 to 1.29)	0.514
Age 55–64†	0.94	(0.63 to 1.39)	0.744
Age 65+†	1.03	(0.7 to 1.5)	0.894
A levels/equivalent‡	0.90	(0.67 to 1.21)	0.493
**Undergraduate degree/equivalent** ‡	**1.43**	(**1.08 to 1.89**)	0.013*
**Postgraduate degree/equivalent** ‡	**1.57**	(**1.13 to 2.18**)	0.007**
Skilled manual workers§	1.05	(0.75 to 1.45)	0.791
Supervisory, clerical and junior managerial, administrative or professional§	1.18	(0.86 to 1.62)	0.295
Higher/intermediate managerial, administrative or professional§	1.18	(0.85 to 1.63)	0.315
IMD	0.99	(0.92 to 1.08)	0.881

Significant relationships (<0.05) are highlighted in bold.

*p<0.05, **p<0.01, ***p<0.001.

* p<0.05, **p<0.01, ***p<0.001.

† Reference category: age 16–24.

‡ Reference category: secondary school or equivalent.

§ Reference category: semiskilled and unskilled manual workers, pensioners, casual and lowest grade workers, unemployed and in receipt of state benefits only.

no/lo, alcohol-free and low-alcohol.

Regarding the sociodemographic characteristics, the analysis revealed a curvilinear relationship between AUDIT-C score (a measure of alcohol use severity) and no/lo alcohol consumption. While AUDIT-C scores were positively linearly associated with an increased likelihood of consuming no/lo drinks at least monthly, the strength of this association weakened at higher levels of AUDIT-C (table 3). Furthermore, compared with the reference group (secondary school education or equivalent), respondents with higher education and postgraduate levels of education were significantly more likely to consume no/lo drinks at least monthly. Sex, age, social grade and IMD were not significant predictors in this model. The AUC was 0.61, indicating fair discrimination in distinguishing between respondents who consume no/lo drinks at least monthly and those who do not.

### Sensitivity analyses

Complete case analysis (n=2118) replicated the primary findings (see online supplemental table 4). When binary classifications of the alcohol drinking motives replaced continuous variables, no significant effects between alcohol drinking motives and at least monthly no/lo consumption were found (online supplemental table 5). Other relationships remained unchanged.

## Discussion

This is the first study to quantitatively explore associations between the reasons adults drink alcohol and the consumption of no/lo drinks. Qualitative research in the UK and Australia has already indicated drinking motives may influence why some people choose to consume no/lo drinks and others do not.^31^,^3336^ If no/lo drinks are promoted to improve public health via substitution, it is important to develop our understanding of how this change may occur.

We hypothesised that those respondents who primarily drank alcohol for internal reasons (enhancement and coping) would be less likely to consume no/lo drinks than those who primarily drank alcohol for other reasons, evident through decreased odds of no/lo consumption among those drinking for these reasons. Drinking for enhancement was associated with an increased rather than decreased odds of drinking no/lo in the unadjusted model. However, this association disappeared once sociodemographic characteristics and hazardous drinking were accounted for, suggesting it was a spurious relationship. In the adjusted model, we found an increased odds of drinking no/lo for those who endorsed drinking alcohol to conform. This corroborates the broader literature, where consumers of no/lo reference the ability to ‘join in’ social occasions, no/lo drinks enabling their ‘non-drinking’ to go un-questioned or un-challenged.^31^,^3335 36^ In line with Perman-Howe *et al*^39^, those who reported higher educational qualifications and higher AUDIT-C scores were also statistically more likely to report drinking no/lo at least monthly. We did not find evidence of an association between neighbourhood level deprivation, measured using the IMD and no/lo consumption.

### Implications for public health and further work

Currently, no/lo drinks are regularly consumed by a minority of adults who drink alcohol. In this study, approximately one-fifth of respondents reported consuming no/lo drinks at least monthly. However, this market is outperforming a declining standard alcohol market.^58^ If consumption increases, there remains potential for no/lo to be of significant public health benefit.

Our study indicates that who may benefit may be contingent on the reasons people drink alcohol in the first place. The regression model results indicate that people who drink to conform are more likely to drink no/lo regularly after accounting for sociodemographic characteristics and hazardous drinking. In the UK, where drinking alcohol is normalised,^59^ no/lo drinks may serve as a welcome alternative for those wishing to reduce their alcohol consumption while circumventing the pressure to conform to the social consensus. However, we must note that the overall effect size was small and the sensitivity analysis which explored drinking motives on a binary scale did not consistently support the associations observed in the primary model. The AUC was 0.61, suggesting that there are other important factors that are associated with no/lo consumption that are not included in this model.

Further work is needed to better understand the nuanced relationship between drinking alcohol to conform and consuming no/lo drinks, particularly among those who are using the drinks as a substitute to standard strength alcohol. The current study explores overall no/lo consumption, including consumption among those who would probably not have been drinking alcohol otherwise, for example, those who are pregnant or driving; and no/lo consumption that does not specifically serve to replace alcohol consumption. Therefore, it is likely the effect of drinking motives among those who are directly substituting is diluted in this study.

It is also important to note that drinking alcohol to conform is typically not one of the most strongly endorsed reasons to drink alcohol at a population level, with just 14% of respondents in this study reporting drinking for this reason at least half of the time. Research indicates that people who predominantly drink for this reason already tend to drink at less harmful levels which may limit the reach of the policy for heavier drinkers drinking alcohol for other reasons.^38^

The most common reasons for drinking alcohol in this study were social and enhancement (table 1), which corresponds with other research of adult alcohol drinking motives in the UK and internationally.^21 60^ Drinking for enhancement is directly associated with heavier drinking, with drinking for social reasons and to cope also directly or indirectly associated with alcohol harms.^38^ If no/lo drinks prove effective for reducing hazardous drinking, it would be important to consider strategies to encourage those who use alcohol as a coping mechanism, for its mood enhancement properties, or to make social occasions more enjoyable to switch to no/lo products, while being mindful that additional approaches may be needed.

Regular consumption of no/lo drinks is positively associated with metrics of social advantage, particularly higher levels of education.^39 61^ Further research is required to understand why this might be. One explanation may be that no/lo drinks are not satisfying the alcohol drinking motives predominant among less advantaged socioeconomic groups, who are more likely to drink alcohol as a coping mechanism than those who are more socioeconomically advantaged.^21 22 46^ Further analysis has explored whether alcohol drinking motives mediate pathways between sociodemographic variables, hazardous drinking and no/lo consumption.^62^

### Strengths and limitations

This study was informed by the qualitative literature on no/lo consumption, which was then mapped onto Cox and Klinger’s motivational model of drinking motives.^24 25 31 33 37^ We used a nationally representative sample of adults aged 16 and over, and living in Great Britain, incorporating sample weights. The items selected, taken from a well-validated scale of drinking motives, were felt to be the most appropriate based on the qualitative literature and were supported by PPI.

A trade-off by using the ATS was that it was not feasible to include the full DMQ-R.^25^ This is not uncommon when using large surveys, where the constructs of interest comprise a small aspect of the survey. Using single items rather than the full scale may limit the validity and reliability of our findings by not fully capturing the dimension it represents. This may have been further compounded by respondents who reported ‘don’t know’ in response to the drinking motive items, whom we excluded from the analysis. If we had chosen different items to represent our constructs, for example, if we had measured enhancement using ‘Because it’s exciting’ rather than ‘Because you like the feeling’, we may have had different findings. However, we took a considered approach to our item selection. The patterns of endorsement for our selected items are consistent with a recently conducted, cross-national study of drinking motives (including Great Britain), supporting the reliability of our estimates.^60^

Due to the cross-sectional design, we are unable to infer causation or explore temporal trends. It was also not possible to explicitly identify whether no/lo drinks are replacing alcoholic beverages. Finally, while representative at the population level, certain at-risk groups are underrepresented in surveys like the ATS, including those residing in care homes, or hospitals, prison inhabitants and the military. It is important to be mindful of this when estimating the impact of this policy on alcohol-specific harms.

## Conclusions

Our study results indicate that regular consumption of no/lo drinks among adults in Great Britain is associated with those who endorse drinking to conform. This aligns with qualitative data on this topic. There was no evidence to suggest a direct association between no/lo consumption and drinking alcohol for how it makes you feel, to make social occasions more enjoyable, or as a coping strategy, once sociodemographic characteristics and alcohol consumption were accounted for. Understanding the potential for benefit of no/lo drinks remains a public health priority, given their inclusion in the incumbent UK government’s 10-year health plan.^5^ The importance of our findings depends on the extent to which no/lo drinks are being used to substitute standard alcoholic drinks. Future work should consider replicating our findings using the full DMQ-R, or similar, exploring the influence of alcohol drinking motives under circumstances where consumers are specifically replacing alcohol with no/lo drinks, and if and how they help to explain sociodemographic differences in consumption.

## Supplementary material

10.1136/bmjph-2025-002828 online supplemental file 1

10.1136/bmjph-2025-002828 online supplemental file 2

## Data Availability

Data may be obtained from a third party and are not publicly available.
