# Supplementary figures and images for: Is why we drink alcohol important when considering the potential public health benefit of alcohol-free and low-alcohol drinks? A cross-sectional study investigating associations between alcohol drinking motives and alcohol-free and low-alcohol drink consumption among adults in Great Britain
Source: BMJ Public Health. 2025 Sep 8;3(2):e002828. doi: 10.1136/bmjph-2025-002828 (PMC12421153; doi:10.1136/bmjph-2025-002828)

**SUPPLEMENTARY FIGURE 1** Participant recruitment and inclusion in the study

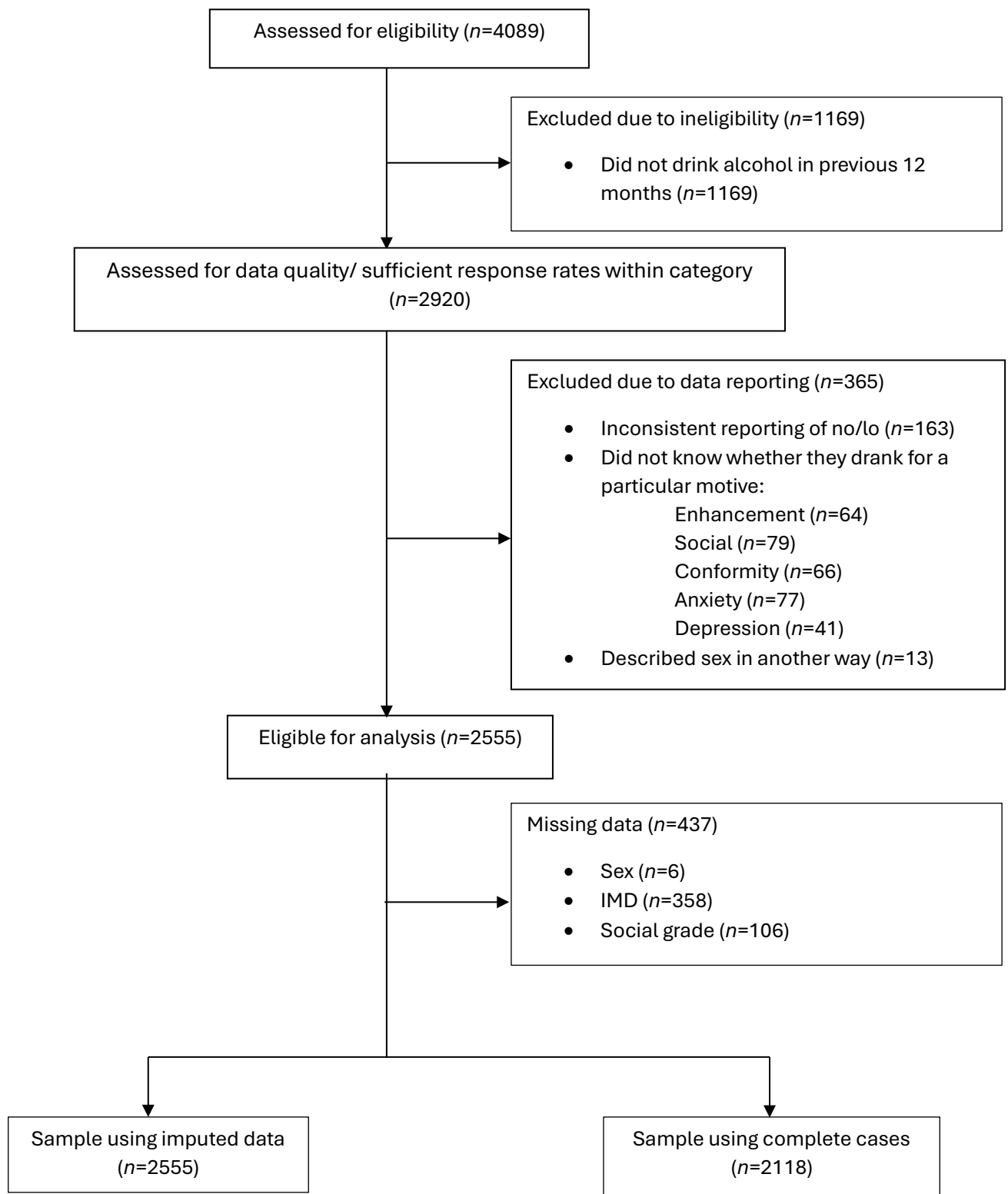

Supplement: online supplemental file 1 [file bmjph-3-2-s001.pdf]
