# Supplementary material for: Is why we drink alcohol important when considering the potential public health benefit of alcohol-free and low-alcohol drinks? A cross-sectional study investigating associations between alcohol drinking motives and alcohol-free and low-alcohol drink consumption among adults in Great Britain
Source: BMJ Public Health. 2025 Sep 8;3(2):e002828. doi: 10.1136/bmjph-2025-002828 (PMC12421153; doi:10.1136/bmjph-2025-002828)
Supplement: online supplemental file 2 [file bmjph-3-2-s002.docx]

**SUPPLEMENTARY MATERIAL**

**SUPPLEMENTARY TABLE 1** An evaluation of the impact of population weighting on the study sample

| **Weighting Metric** |  | **Value** | |
| --- | --- | --- | --- |
| Weight Distribution (Range) | | 0.27 – 3.20 | |
| Initial Sample Size (N) | | 2555 | |
| Effective Sample Size (ESS) | | 2053 | |
| Design Effect (DEFF) | | 1.24 | |
| **Variable** | **Category** | **Unweighted %** | **Weighted % (SE)** |
| Gender | Men | 53.2% | 51% (1.1) |
|  | Women | 46.8% | 49% (1.1) |
| Social Grade | DE | 13.5% | 19.2% (1) |
|  | C2 | 14.6% | 21.5% (1) |
|  | C1 | 42.9% | 29.1% (0.9) |
|  | AB | 29% | 30.1% (1) |
| Education | Secondary School/equivalent | 24% | 25.9% (1) |
|  | Further education 16+/equivalent | 24.9% | 25.7% (1) |
|  | Undergraduate degree/ equivalent | 33.1% | 31.7% (1) |
|  | Post-graduate degree/ equivalent | 18% | 16.7% (0.8) |
| AUDIT-C | Mean | 4.39 | 4.36 (0.06) |
| IMD | Mean | 3.2 | 3.12 (0.03) |

**SUPPLEMENTARY TABLE 2** Testing for multicollinearity among independent variables using Variance Inflation Factors (VIFs)

| **Indicator** | **Odds Ratio** | **95% CI** | **p value** | **VIF** |
| --- | --- | --- | --- | --- |
| (Intercept) | 0.08 | (0.04, 0.14) | 0.000 |  |
| Enhancement | 1.03 | (0.95, 1.11) | 0.532 | 1.34 |
| Social | 0.94 | (0.86, 1.02) | 0.131 | 1.50 |
| Conformity | 1.10 | (1, 1.21) | 0.041 | 1.23 |
| Anxiety | 1.09 | (0.99, 1.21) | 0.092 | 1.40 |
| Depression | 1.02 | (0.9, 1.15) | 0.799 | 1.21 |
| AUDIT-C (linear) | 1.41 | (1.21, 1.64) | 0.000 | 15.22 |
| AUDIT-C (quadratic) | 0.98 | (0.96, 0.99) | 0.000 | 14.92 |
| Women (compared to men) | 0.86 | (0.71, 1.05) | 0.149 | 1.07 |
| Age 25-34^a^ | 0.94 | (0.64, 1.38) | 0.756 | 1.34 |
| Age 35-44^a^ | 1.00 | (0.68, 1.47) | 0.985 | 1.34 |
| Age 45-54^a^ | 0.88 | (0.6, 1.29) | 0.514 | 1.34 |
| Age 55-64^a^ | 0.94 | (0.63, 1.39) | 0.744 | 1.34 |
| Age 65+^a^ | 1.03 | (0.7, 1.5) | 0.894 | 1.34 |
| A levels / equivalent^b^ | 0.90 | (0.67, 1.21) | 0.493 | 1.38 |
| Undergraduate degree/ equivalent^b^ | 1.43 | (1.08, 1.89) | 0.013 | 1.38 |
| Postgraduate degree/ equivalent^b^ | 1.57 | (1.13, 2.18) | 0.007 | 1.38 |
| Skilled manual workers^c^ | 1.05 | (0.75, 1.45) | 0.791 | 1.32 |
| Supervisory, clerical and junior managerial, administrative or professional^c^ | 1.18 | (0.86, 1.62) | 0.295 | 1.32 |
| Higher/intermediate managerial, administrative or professional^c^ | 1.18 | (0.85, 1.63) | 0.315 | 1.32 |
| IMD | 0.99 | (0.92, 1.08) | 0.881 | 1.08 |

Reference cases: ^a^ Age 16-24, ^b^ No formal qualifications, ^c^ Semi-skilled and unskilled manual workers, pensioners, casual and lowest grade workers, unemployed and in receipt of state benefits only.

Interpretation Guide for VIFs:

- VIF = 1: No multicollinearity

- VIF < 5: Generally acceptable

- VIF > 5: Potential multicollinearity issues

- VIF > 10: Severe multicollinearity issues

Note: The 'structural multicollinearity' between the audit variables does not violate the assumptions of the regression model.

**SUPPLEMENTARY TABLE 3** The percentage of low and high endorsers of each alcohol drinking motive who reported regular no/lo consumption, with 95% Confidence Intervals (weighted, *n*=2597)

| **Drinking Motive** | **Endorsement Level** | **Percentage (%)** | **Lower 95% CI** | **Upper 95% CI** |
| --- | --- | --- | --- | --- |
| Enhancement | less than half the time | 19.1 | 16.9 | 21.4 |
| Enhancement | at least half the time | 23.7 | 21.0 | 26.4 |
| Social | less than half the time | 19.9 | 17.7 | 22.1 |
| Social | at least half the time | 22.9 | 20.1 | 25.7 |
| Conformity | less than half the time | 20.5 | 18.7 | 22.4 |
| Conformity | at least half the time | 25.4 | 20.4 | 30.3 |
| Anxiety | less than half the time | 20.3 | 18.5 | 22.1 |
| Anxiety | at least half the time | 26.5 | 21.2 | 31.8 |
| Depression | less than half the time | 21.2 | 19.4 | 23.0 |
| Depression | at least half the time | 20.5 | 13.4 | 27.5 |

**SUPPLEMENTARY TABLE 4** Results from the sensitivity analysis using complete data (weighted)

| **Indicator** | **Odds Ratio** | **95% CI** | **p value** |
| --- | --- | --- | --- |
| (Intercept) | 0.09 | (0.04, 0.17) | 0.000 |
| Enhancement | 1.04 | (0.95, 1.13) | 0.412 |
| Depression | 0.97 | (0.83, 1.12) | 0.656 |
| Social | 0.91 | (0.83, 1) | 0.051 |
| **Conformity** | **1.12** | **(1.01, 1.25)** | **0.033*** |
| Anxiety | 1.03 | (0.91, 1.16) | 0.636 |
| **AUDIT-C (linear)** | **1.48** | **(1.25, 1.75)** | **0.000***** |
| **AUDIT-C (quadratic)** | **0.97** | **(0.96, 0.99)** | **0.000***** |
| Women (compared to men) | 0.86 | (0.69, 1.08) | 0.193 |
| Age 25-34^a^ | 0.91 | (0.58, 1.42) | 0.670 |
| Age 35-44^a^ | 0.93 | (0.6, 1.45) | 0.746 |
| Age 45-54^a^ | 0.77 | (0.49, 1.21) | 0.261 |
| Age 55-64^a^ | 0.81 | (0.51, 1.27) | 0.354 |
| Age 65+^a^ | 0.91 | (0.58, 1.41) | 0.665 |
| A levels / equivalent^b^ | 0.98 | (0.7, 1.37) | 0.911 |
| **Undergraduate degree/ equivalent**^b^ | **1.58** | **(1.16, 2.17)** | **0.004**** |
| **Postgraduate degree/ equivalent**^b^ | **1.77** | **(1.23, 2.54)** | **0.002**** |
| Skilled manual workers^c^ | 1.09 | (0.75, 1.57) | 0.650 |
| Supervisory, clerical and junior managerial, administrative or professional^c^ | 1.15 | (0.81, 1.63) | 0.423 |
| Higher/intermediate managerial, administrative or professional^c^ | 1.21 | (0.85, 1.73) | 0.299 |
| IMD | 0.97 | (0.9, 1.06) | 0.525 |

Significant relationships (<.05) are highlighted in bold

*p <.05, *** p <.001

Reference cases: ^a^ Age 16-24, ^b^ No formal qualifications, ^c^ Semi-skilled and unskilled manual workers, pensioners, casual and lowest grade workers, unemployed and in receipt of state benefits only.

**SUPPLEMENTARY TABLE 5** Results from the sensitivity analysis using binary endorsement levels for alcohol drinking motives (weighted)

| **Indicator** | **Odds Ratio** | **95% CI** | **p value** |
| --- | --- | --- | --- |
| (Intercept) | 0.09 | (0.05, 0.16) | 0.000 |
| High vs low endorsers Enhancement | 1.05 | (0.85, 1.31) | 0.643 |
| High vs low endorsers Social | 0.96 | (0.77, 1.21) | 0.734 |
| High vs low endorsers Conformity | 1.18 | (0.88, 1.57) | 0.273 |
| High vs low endorsers Anxiety | 1.33 | (0.99, 1.79) | 0.060 |
| High vs low endorsers Depression | 0.84 | (0.55, 1.29) | 0.423 |
| **AUDIT-C (linear)** | **1.39** | **(1.19, 1.62)** | **0.000** |
| **AUDIT-C (quadratic)** | **0.98** | **(0.96, 0.99)** | **0.001** |
| Women (compared to men) | 0.87 | (0.71, 1.06) | 0.163 |
| Age 25-34^a^ | 0.95 | (0.65, 1.4) | 0.801 |
| Age 35-44 ^a^ | 1.02 | (0.69, 1.49) | 0.935 |
| Age 45-54 ^a^ | 0.89 | (0.61, 1.31) | 0.563 |
| Age 55-64 ^a^ | 0.95 | (0.64, 1.41) | 0.807 |
| Age 65+ ^a^ | 1.04 | (0.72, 1.52) | 0.825 |
| A levels / equivalent^b^ | 0.89 | (0.66, 1.19) | 0.424 |
| **Undergraduate degree/ equivalent^b^** | **1.40** | **(1.06, 1.86)** | **0.018** |
| **Postgraduate degree/ equivalent^b^** | **1.55** | **(1.12, 2.14)** | **0.009** |
| Skilled manual workers^c^ | 1.04 | (0.75, 1.44) | 0.828 |
| Supervisory, clerical and junior managerial, administrative or professional^c^ | 1.17 | (0.85, 1.6) | 0.330 |
| Higher/intermediate managerial, administrative or professional^c^ | 1.17 | (0.85, 1.62) | 0.338 |
| IMD | 0.99 | (0.92, 1.08) | 0.877 |

Significant relationships (<.05) are highlighted in bold

* p <.05, ** p <.01, ***p<.001

Reference cases: ^a^ Age 16-24, ^b^ No formal qualifications, ^c^ Semi-skilled and unskilled manual workers, pensioners, casual and lowest grade workers, unemployed and in receipt of state benefits only.
